# Report of a Case of Puerperal Convulsions, with Remarks on the Use of Chloroform

**Published:** 1855-05

**Authors:** William Clarke


THE NORTH-WESTERN

MEDICAL AND SURGICAL JOURNAL.

NEW SERIES.

VOL. IV. MAY, 1855. NO. 5.

### ORIGINAL COMMUNICATIONS

ART. I.—*Report of a case of Puerperal Convulsions, with remarks on the use of Chloroform.* By WM. CLARKE, M.D., read to the Cook County Medical Society, Chicago, April 3d, 1855.

Mrs. B. an English woman of small stature, square form, and full habit, aged twenty years, the wife of a laborer—became pregnant for the first time, and enjoyed the best of health through the period of gestation, until within two or three days of her confinement. During these days she was troubled with pain in her head, which, on the morning of her confinement was exceedingly severe. At this time her vision must have been impaired, as she complained to those around her, of its being dark.

A midwife was called in, and at one o'clock P. M., on the 19th of November, 1854, the patient was delivered of a full grown healthy child, having, as was expressed by the woman in attendance, "an easy time." A few moments after delivery, she went into a succession of convulsions.

The intervals were from ten to thirty minutes duration, marked by stertorous breathing, and perfect loss of consciousness. It was in this state that I first saw her, at 4½ o'clock, P. M. She was soon after taken with a poroxysm, so violent in its character, that the attendants could barely confine her to the bed. The swollen veins of the head and neck—the livid hue of the countenance—the blood-shot eyes—the tongue protruding from the mouth, and badly lacerated by the violent closure of the jaws, the agonizing contortions of the whole body, presented all the symptoms of a most frightful attack of epilepsy. The pulse was full, hard and quick. After getting a cork between the teeth, to relieve the tongue, a vein was opened, and when about ten ounces of blood had escaped, the paroxysm ceased, the pulse bec me soft, and less frequent, and the breathing much more natural than in the preceding interval. Mixed thirty grs. of calomel with treacle, placed it in her mouth, and held her nose until she swallowed it. Applied dry cups to the nape of neck and back, and ordered sinapisms to extremities.

The calm continued twenty minutes, when another fit came on, but much more mild than the preceding.

At this time, owing to the absence of the midwife, and the women who were with the patient during labor, I was unacquainted with the history of the case, and of course, knew nothing of the marked head symptoms that had existed from the first signs of labor, the account of which I obtained some hours after. Knowing that chloroform was greatly extolled by gentlemen, eminent in the profession, I was induced to give it a trial, I mixed some thirty drops with treacle, and endeavored to get the patient to swallow it. But only partly succeeding in that, I put sixty drops on a napkin, and applied to the mouth and nostrils. The only apparent effect was the decided aggravation of all the worst symptoms for the remainder of the paroxysm, and when the calm at last succeeded, the interval was much shorter, and the coma far more profound.

The chloroform was discontinued, and by injections of salt and water per anum—cold to the head, and all the means within reach, efforts were made to divert the blood into other parts of the system, where its presence would be less dangerous, than on the brain and spinal cord, but no favorable impression on the disease was apparent. At 10 o'clock P. M., another orifice was made in the arm. and the blood flowed until the character of the pulse and the color of the lips and countenance had decidedly changed. There was a marked mitigation of all the symptoms.

The patient being able to swallow without any difficulty, an ounce of castor oil was administered, and a quarter of a grain tart. ant. and potass. The latter, to be repeated every hour.

On the 20th at 7 o'clock A. M., the convulsions were less violent, with much longer intervals, during which, the breathing appeared more natural, The b wels had not moved, and not the least discharge from the vagina; skin was hot and dry; pulse hard and frequent; the *pupil* of the eye dilated; *intolerance* of *light.* Applied blisters to the nape of the neck, and behind the ears; cold to the head; excluded the light from the room; administered three of the comp cathartic pills, and continued the tartar emetic.

At 12 o'clock, P. M; convulsions had ceased; consciousness partially restored. The patient could answer questions, and readily swallowed drink when offered, but was evidently maniacal, repelling those who offered to move her, most furiously. There was *intolerance* of *light* and decided amaursis. The skin, circulation and bowels as before. Continued, tart emetic and gave a solution of camphor in sul. ether to allay restlessness, and directed two drops of croton oil, and thirty drops of turpentine in emulsion, to be given every two hours.

At 7 o'clock P. M the patient had taken three doses of the emulsion, and there had just been a slight discharge from the bowels, and the symptoms had a good deal improved.

Nov. 21st.—The patient had several discharges from the bowels during the night, and appeared quite rational and perfectly relieved. The skin was bathed in a gentle perspiration.

Had not the least recollection of anything that had passed since the morning of the 19th inst. Could hardly believe that she was a mother.

Nov 2d.—Continued to improve; a good deal of soreness of the tongue, and quite weak; otherwise very comfortable.

Convalescence rapid and uninterrupted.

In glancing at the prominent points in this case, the question naturally arises, was the administration of chloroform proper? For one, I must candidly admit that it was not, and my only excuse for prescribing it was the many respectable authorities in its favor. Subsequently, from having my attention particularly directed to the subject, the question has arisen in my own mind whether the medicine should be given in any case, or in any stage of puerperal convulsions, except in those forms of hysterical convulsions, which may come on at any time during gestation, or in childhood, but which more commonly arise during the former period. In this form of disease, any remedy of the kind could, and most probably would, be used with advantage.

Is not congestion of the head and neck a very common, if not one of the most obvious effects of the inhalation of chloroform or ether? And would not this effect be produced by the vapor, to a certain extent, taking the place of atmospheric air in respiration, aside from the direct influence the medicine might exert on the circulation? In a disease so constantly marked by a determination of blood to the head, that copious bleeding has always been, and probably will ever be, considered the most prompt and perfect remedy, can the employment of an agent that has a tendency to produce the same state of things be in accordance with sound Therapeutics?

Again, it is not uncommon for chloroform to produce twitching of the muscles and frothing of the mouth, symptoms analogous to epilepsy.

It was only last week, in administering chloroform while my my friend Dr. Miller performed a painful operation, that the patient was taken with convulsions as soon as he began to feel the influence of the anæsthetic. The dread of the operation had, doubtless, some agency in producing the peculiar state of the nervous system. But that alone would not have given rise to spasms. It might have been this view of the subject that led Prof. Bache to say, in these very words: "It is a good rule *not* to administer chloroform to persons subject to epilepsy."

If it does have the tendency we are speaking of, would not the giving of it, then, in such a disease, be too much in keeping with the vagaries of homœopathy, and too little in accordance with the dictates of a rational philosophy?

[Dr. J. V. Z. Blaney doubted whether the inhalation of chloroform produced fulness of the vessels of the brain and spinal cord, or a congestion in these parts. He said experience had shown that the *recumbent* posture was the best and safest for the patient while receiving the chloroform; that when fully under its influence, the face is more frequently pale than flushed; the carotids soft than full and hard; and when the dose administered becomes excessive, all the symptoms are those resembling *syncope*, instead of fulness or congestion. So, too, the remedies most effectual for counteracting the effects of an over dose are the same as for syncope. Dr. B. thought anæsthesia or insensibility to pain was by no means always produced by excessive accumulation of carbon or deficiency of oxygen in the blood; that the inhalation of chloroform did not, like that of ether, materially increase the quantity of carbon in the blood; but that any agent capable of arresting the processes on which *innervation* depends, would prove anæsthetic, even though it might introduce an excess of oxygen into the system, as was the case when nitrous oxide gas was inhaled.

Dr. D. Miller thought the effects of inhaling chloroform were very much influenced by the method adopted. He had never seen bad effects produced when it was inhaled slowly, and with a free admixture of atmospheric air. But thought a too rapid inhalation very liable to produce spasmodic action and dangerous symptoms.

Dr. Hitchcock said he had not observed any symptoms of increased fulness of the vessels of the neck and head in patients under the influence of chloroform.

Dr. Bloodgood agreed with Dr. Blaney, that anæsthesia could not depend, in all cases at least, on diminished decarbonization of the blood.

Dr. Blaney thought the inhalation of chloroform one of the most prompt and beneficial remedies we possess for puerperal convulsions of a hysterical character, but should not regard it as safe in those cases partaking of the nature of apoplexy. But he acknowledged that the diagnosis between the two varieties was not always easy.

Dr. H. Smith did not see clearly why chloroform should be injurious in apoplectic cases, if it lessened rather than increased the fulness of the brain.—N. S. D.]

